# Chemical Stability of Ti_3_C_2_ MXene with Al in the Temperature Range 500–700 °C

**DOI:** 10.3390/ma11101979

**Published:** 2018-10-15

**Authors:** Jing Zhang, Shibo Li, Shujun Hu, Yang Zhou

**Affiliations:** Centre of Materials Science and Engineering, School of Mechanical and Electronic Control Engineering, Beijing Jiaotong University, Beijing 100044, China; 16121356@bjtu.edu.cn (J.Z.); 16116341@bjtu.edu.cn (S.H.); yzhou@bjtu.edu.cn (Y.Z.)

**Keywords:** Ti_3_C_2_T_x_, MXene, aluminum, chemical stability, microstructure

## Abstract

Ti_3_C_2_T_x_ MXene, a new 2D nanosheet material, is expected to be an attractive reinforcement of metal matrix composites because its surfaces are terminated with Ti and/or functional groups of –OH, –O, and –F which improve its wettability with metals. Thus, new Ti_3_C_2_T_x_/Al composites with strong interfaces and novel properties are desired. To prepare such composites, the chemical stability of Ti_3_C_2_T_x_ with Al at high temperatures should be investigated. This work first reports on the chemical stability of Ti_3_C_2_T_x_ MXene with Al in the temperature range 500–700 °C. Ti_3_C_2_T_x_ is thermally stable with Al at temperatures below 700 °C, but it reacts with Al to form Al_3_Ti and TiC at temperatures above 700 °C. The chemical stability and microstructure of the Ti_3_C_2_T_x_/Al samples were investigated by differential scanning calorimeter, X-ray diffraction analysis, scanning electron microscopy, and transmission electron microscopy.

## 1. Introduction

MXenes, as a new family of two-dimensional (2D) materials, have attracted much attention because of their unusual combination of mechanical, physical, and chemical properties [1,2,3,4,5,6,7,8,9,10]. The MXenes are produced by the selective extraction of A-element from the layered MAX phases (where M is an early transition metal, A is an A group element, and X is C or N), such as Ti_3_AlC_2_, Ti_2_AlC, and Ti_3_SiC_2_ by either HF solutions [1,2,11,12,13], HCl/fluoride salt solutions [3,14,15,16,17,18], or HF/oxidant solutions [19]. After removal of the A layers from MAX, the top and bottom surfaces of the bare MXene monolayer or single sheet are terminated by the redistribution of M atoms with metallic bonds. Hence, the MXenes exhibit metallic conductivity. However, the exposed M surfaces of MXenes are always attached by functional groups such as hydroxyl (–OH), oxide (–O), or fluorine (–F) after etching in acid solutions. Hence, the general formula M_n+1_X_n_T_x_ (where T_x_ denotes the MXene terminal groups and x is number of terminal groups, n = 1, 2, or 3) is always used to represent the MXenes.

MXenes have similar structure and properties to graphene and other 2D nanosheets. The attractive 2D MXenes are now being used in a wide range of applications in energy storage, electromagnetic interference shielding, water purification, gas- and biosensors, lubrication, photo-, electro- and chemical catalysis, and as reinforcement for composites [3,5,6,7,12,20,21,22,23,24,25,26,27,28,29,30].

In the 2D MXene group, Ti_3_C_2_T_x_ is the first reported and the most explored material because it is easily synthesized from the Ti_3_AlC_2_ precursor using HF solution [1]. The 2D Ti_3_C_2_T_x_ single nanosheet is micrometers in width but less than a nanometer in thickness. Hence, the Ti_3_C_2_T_x_ MXene, with its attractive properties and high aspect ratios, has already been used as a reinforcement in composites. So far, the majority of the current work on composites containing Ti_3_C_2_T_x_ has focused on polymer matrix composites [30,31,32,33,34], but less attention has been devoted to the use of Ti_3_C_2_T_x_ to reinforce metal matrix composites.

In recent years, there has been intense research into Al matrix composites reinforced with 1D carbon nanotubes (CNTs) and 2D graphene to improve their mechanical properties [35,36,37,38]. However, there remains a challenge to overcome weak interfaces in the CNT/Al and graphene/Al composites, due to the poor wettability of CNTs and graphene with Al matrix.

Using Ti_3_C_2_T_x_ to reinforce the Al metal matrix composites possibly endows the Ti_3_C_2_T_x_/Al composites with a strong interfacial strength because the surfaces of Ti_3_C_2_T_x_ nanosheets are terminated with Ti or functional groups such as –OH, –O, and –F. It has been reported that the surface modification of CNTs by –O and –OH functional groups enhanced the interfacial bonding between metals and CNTs [39,40]. The functionalized 2D nanotubes can be employed as reinforcing fillers [41,42]. In addition, the –F and –O surface functional groups on the Ti_3_C_2_T_x_ nanosheets can be eliminated at high temperatures [43,44]. The loss of surface functional groups possibly promotes the bonding of Ti surface atoms on bare Ti_3_C_2_ with Al. However, work on the Ti_3_C_2_T_x_/Al composites has been much less focused so far. The ability to practically produce Ti_3_C_2_T_x_/Al composites remains to be demonstrated. Al matrix composites reinforced with 1D CNTs and 2D graphene have always been prepared in the temperature range 500–640 °C. To practically prepare Ti_3_C_2_T_x_/Al composites and to understand the chemical stability of Ti_3_C_2_T_x_ with Al, the temperature range 500–700 °C was selected in the present study.

The purpose of this work was to assess the chemical stability of Ti_3_C_2_T_x_ with Al in the temperature range from 500 °C to 700 °C by X-ray diffraction, differential scanning calorimetry, scanning electron microscopy, and transmission electron microscopy methods. The interfaces between Ti_3_C_2_T_x_ and Al were characterized.

## 2. Materials and Methods

Ti_3_C_2_T_x_ MXene was prepared by acid etching of Ti_3_AlC_2_ powder. The fabrication of Ti_3_AlC_2_ has been described elsewhere [45]. Briefly, Ti (325-mesh, >99.2% purity), Al (particle size <5 μm, 99.5% purity), and C (graphite, <45 µm, >99.5% purity) powders with a molar ratio of Ti:Al:C = 3:1.1:2 were mixed for 10 h. The mixed mixture was cold-pressed to form compacts with a diameter of 50 mm and a height of approximately 5 mm. The compacts were pressurelessly sintered at 1450 °C for 1 h in an Ar atmosphere. The sintered samples were pulverized and then sifted with a 300-mesh sieve to make Ti_3_AlC_2_ powder. A total of 2.5 g of Ti_3_AlC_2_ powder was immersed in 60 mL of 40% HF solution in a polytetrafluoroethylene (PTFE) container. A heating magnetic stirrer was used to continuously stir the solution in the PTFE container at 50 °C for 0.5 h. The solution was centrifugally separated in a centrifuge (LD-4, Jinan Wohong Experimental instrument Co., Ltd., Jinan, China) with a rotation rate of 4000 rpm for 5 min and then washed with deionized water until a pH of about 7 was attained. The obtained sediment was vacuum dried at 80 °C for 24 h to make the desired Ti_3_C_2_T_x_ powder. The prepared Ti_3_C_2_T_x_ powder was sieved with a 200-mesh sieve.

Al and 10 wt % Ti_3_C_2_T_x_ powders were mixed in a polypropylene container with agate balls for 10 h in a rotary drum type ball-miller with a speed of 150 rpm. The mixture was cold-pressed in a stainless steel mold with 100 MPa to form pellets with a diameter of 20 mm and a height of about 5 mm. The pellets were put into a graphite crucible coated with boron nitride and then sintered in the absence of additional pressure conditions in the temperature range 500–700 °C for 1 h in Ar.

The phase composition of the samples before and after sintering was identified by X-ray diffraction (XRD) analysis using a D/Max 2200 PC diffractometer (Rigaku Co. Ltd., Tokyo, Japan) applying monochromatic Cu Kα radiation. The operating voltage and current were 40 kV and 20 mA, respectively. The microstructures of the sintered samples were characterized with a ZEISS EVO 18 scanning electron microscope (SEM) equipped with an energy-dispersive spectrometer system (EDS), and a JEM-2100F (JEOL Ltd., Tokyo, Japan) transmission electron microscope (TEM) with an operating voltage of 200 kV. TEM images were processed with the software RADIUS Desktop 2.0 (EMSIS GmbH, Muenster, Germany). Differential scanning calorimetry (DSC) analysis was used to measure the amount of energy absorbed or released by the Ti_3_C_2_T_x_/Al mixture heated in a NETZSCH STA 449F3 thermal analyzer (Netzsch, Germany) from room temperature to 800 °C with a heating rate of 15 °C/min in flowing Ar.

## 3. Results and Discussion

**Figure 1:**
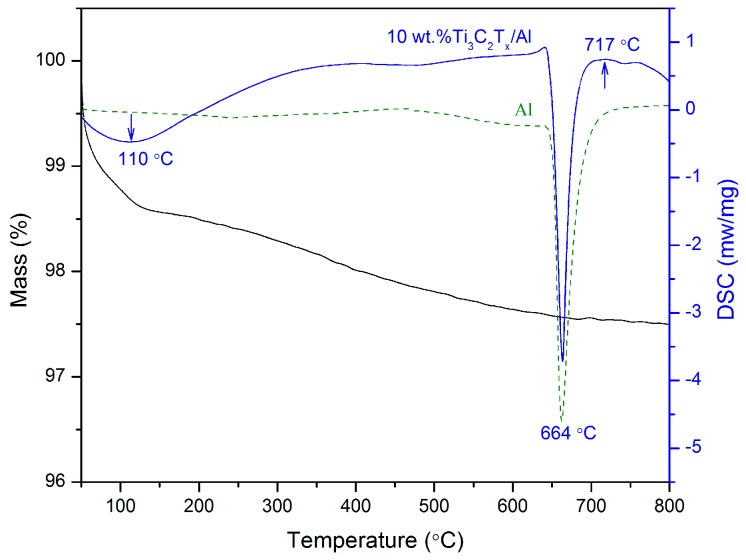
DSC analysis for 10 wt % Ti_3_C_2_/Al mixture in the temperature range 50–800 °C, together with Al for comparison.

The DSC curve of the Ti_3_C_2_T_x_/Al mixture, together with that of pure Al for comparison, is presented in Figure 1. A broad endothermic peak appearing at around 110 °C on the DSC curve for Ti_3_C_2_T_x_/Al is ascribed to the evaporation of water absorbed in the interlayers of Ti_3_C_2_T_x_. The thermal gravity (TG) curve correspondingly exhibits a downward trend on mass loss. A sharp endothermic peak at 664 °C corresponds to the melting of Al (660 °C). Comparing the two DSC curves, it can be found that there is an exothermic peak at 717 °C on the DSC curve of Ti_3_C_2_T_x_/Al, which can be ascribed to the occurrence of a reaction between Ti_3_C_2_T_x_ and Al. In the temperature range from 150 °C to 700 °C, the TG curve reveals a gradual mass loss, possibly due to the removal of surface groups such as –OH and –O. Zhou et al. [43] reported that there was a broad and weak exothermic peak in the DSC curve for the pure Ti_3_C_2_T_x_ in the temperature range 200–800 °C in Ar due to the loss of surface groups. Shah et al. [46] reported that the bonding energies for –O, –OH, and –F groups in the Ti_3_C_2_T_x_ MXene are 530.3 eV, 531.3 eV, and 685.1 eV, respectively. The bonding energies suggest that the removal sequence of the surface groups is –O > –OH > –F. Li and coworkers [44] reported that the disappearance of –OH and –O groups occurred at approximately 500 K and the removal of –F group was required at a high temperature of 1173 K in Ar. Sang and coworkers also confirmed that the functional group of –O was removed after annealing of Ti_3_C_2_T_x_ MXene at 500 °C [47].

To identify the reaction products and to characterize the microstructure, the samples sintered at temperatures from 500 °C to 700 °C were examined using XRD, SEM, and TEM.

**Figure 2:**
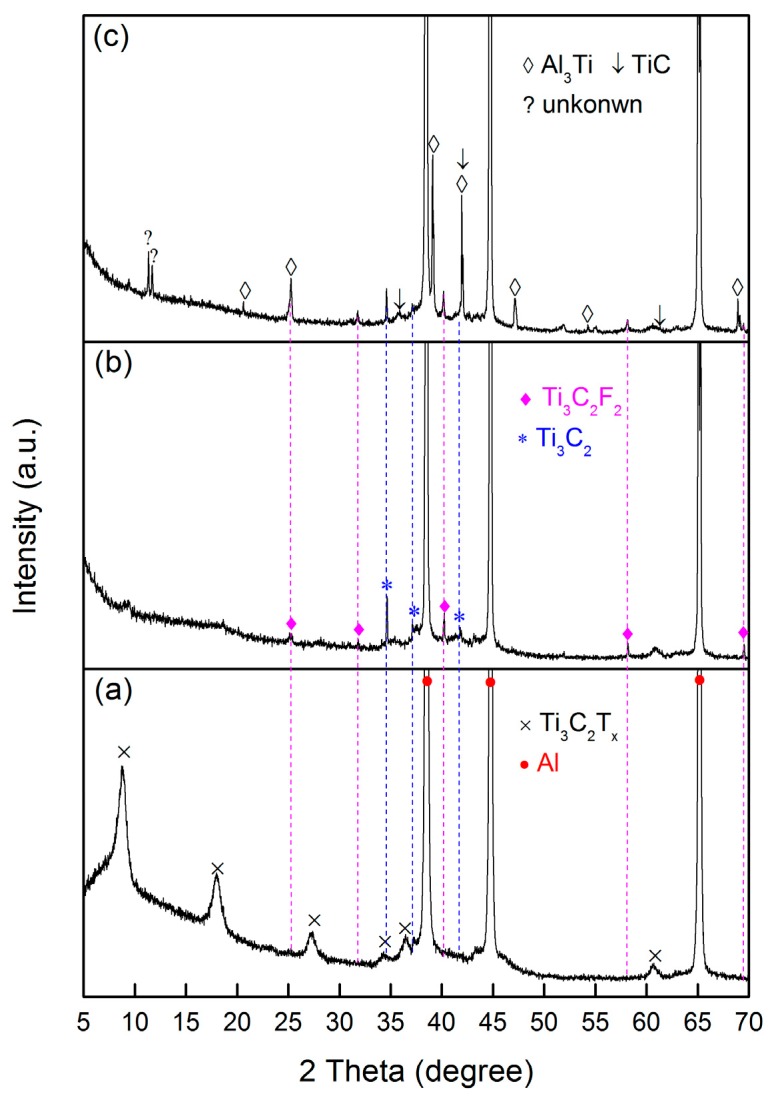
XRD patterns of the Ti_3_C_2_/Al mixture before (**a**), and after heat treatment at (**b**) 650 °C and (**c**) 700 °C in Ar for 1 h.

Figure 2 shows XRD patterns of 10 wt % Ti_3_C_2_T_x_/Al samples before and after sintering at various temperatures. Before sintering, only Ti_3_C_2_T_x_ MXene and Al are detected, see Figure 2a. The broad peaks indicate the nanosheet structure of Ti_3_C_2_T_x_. After sintering at 650 °C, some sharp diffraction peaks but with lower intensities appeared, in addition to the peaks corresponding to Al. These peaks are in good agreement with the simulated peaks for pure Ti_3_C_2_ and Ti_3_C_2_F_2_, see Figure 2b. It should be noted that the (002) peak at 8.7°, (004) peak at 17.98°, and (006) peak at 27.38° belonging to the Ti_3_C_2_T_x_ MXene disappeared in the XRD patterns of the sintered samples, see Figure 2b,c. The XRD results suggest that water molecules associated with the interlayer functional groups such as –O and –OH were gradually removed from 100 °C to 650 °C. The loss of water and functional groups causes a decrease in the basal space distance, or, alternatively, the newly formed surfaces possibly bond to form a new structure, which potentially induces the disappearance of basal peaks. The disappearance of basal plane peaks is similar to those for some layered compounds such as montmorillonite [48] and tetratitanate [49]. However, the diffractogram of the sample sintered at 700 °C shows the appearance of new phases of Al_3_Ti and TiC, indicating the occurrence of a reaction between Ti_3_C_2_T_x_ and Al at 700 °C. This result is in good agreement with the DSC analysis. Some weak peaks belonging to unreacted Ti_3_C_2_ and Ti_3_C_2_F_2_ can still be found. However, several peaks appearing in Figure 2c were not confirmed.

**Figure 3:**
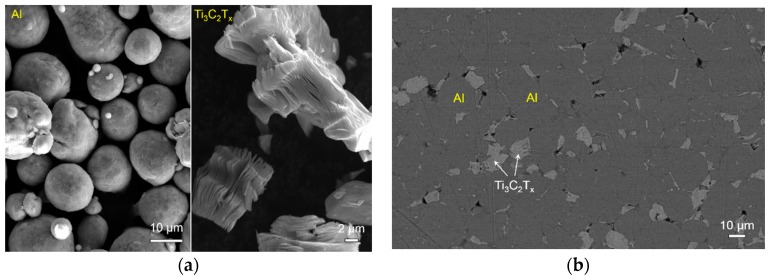

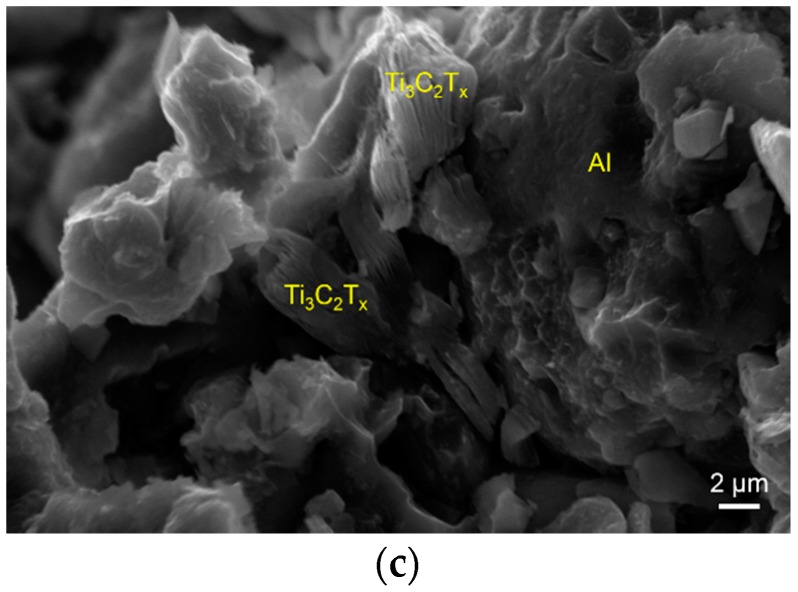
SEM micrographs of the initial powders (**a**), and polished surface (**b**) and fracture surface (**c**) of the samples sintered at 650 °C in Ar for 1 h. The left- and right-hand side micrographs in (**a**) show the morphologies of Al and Ti_3_C_2_T_x_ powders, respectively.

Figure 3 presents the morphologies of the raw Al and Ti_3_C_2_T_x_ powders and microstructures of the sintered samples. The raw Al particles are spherical, and each Ti_3_C_2_T_x_ particle has stacked multilayers, as shown in Figure 3a. After sintering at 650 °C, the polished surface shows that the Ti_3_C_2_T_x_ particles mainly distribute at the Al grain boundaries, see Figure 3b. No other new phases were detected in the sample sintered at 650 °C. The fracture surface clearly shows the stacked multilayers of Ti_3_C_2_T_x_ particles, see Figure 3c.

**Figure 4:**
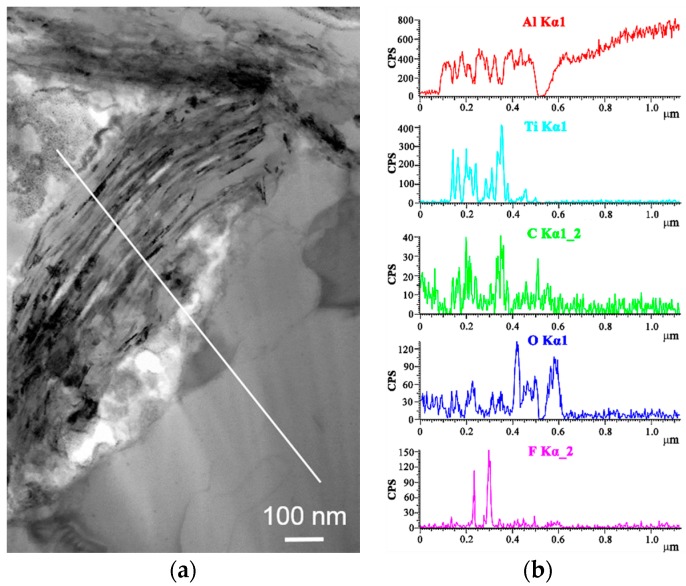
TEM image (**a**) and EDS results of the Ti_3_C_2_T_x_/Al sample sintered at 650 °C (**b**). The EDS scan analysis along the line in (**a**) showing the elemental distribution of Al, Ti, C, O, and F.

Figure 4a presents a TEM image of a multilayer Ti_3_C_2_T_x_ flake. The EDS line analysis, see Figure 4b, reveals the Al signal in the Ti_3_C_2_T_x_ flake, indicating the diffusion of Al atoms into the multilayers of Ti_3_C_2_T_x_. The interlayer spacing areas of Ti_3_C_2_T_x_ provide active sites for Al nucleation and crystal growth. It should be noted that the F element was detected in the Ti_3_C_2_T_x_ multilayers, but O was mainly distributed in the areas between the Ti_3_C_2_T_x_ flake and Al grains. The presence of F in the multilayers suggests that the surface-layer termination of MXene is mostly F, i.e., Ti_3_C_2_F_2_. The –O group in Ti_3_C_2_T_x_ may react with Al to form Al_2_O_3_.

**Figure 5:**
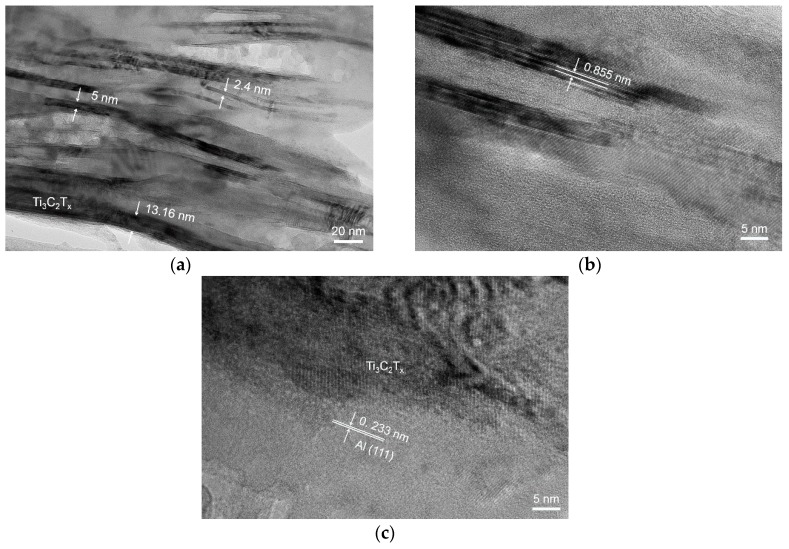
TEM images of the sample sintered at 650 °C. (**a**) A TEM micrograph showing stacked multilayers of Ti_3_C_2_T_x_, (**b**) a high-resolution TEM (HRTEM) micrograph of Ti_3_C_2_T_x_, and (**c**) a HRTEM micrograph of Ti_3_C_2_T_x_/Al interface.

Figure 5 presents the TEM micrographs of a Ti_3_C_2_T_x_/Al sample sintered at 650 °C. These Ti_3_C_2_T_x_ flakes are from several nanometers to tens of nanometers in thickness, indicating the thinner flake is composed of at least two Ti_3_C_2_T_x_ layers, see Figure 5a. The interspaces in the Ti_3_C_2_T_x_ flakes are filled with Al. A high-resolution TEM (HRTEM) image shows that the interlayer spacing of Ti_3_C_2_T_x_ is about 0.855 nm, see Figure 5b. This value is smaller than the value of 1.17 to 1.28 nm for the initial Ti_3_C_2_T_x_ flakes due to the removal of functional groups after heat treatment at 650 °C. The HRTEM image of the Ti_3_C_2_/Al interface reveals that the lattices of the Ti_3_C_2_ and Al regions are in direct contact, as shown in Figure 5c. The interface is clean and continuous. Neither precipitates nor amorphous regions are observed at the interface. The above observation suggests that the Ti_3_C_2_/Al interface is chemically and structurally stable at 650 °C.

## 4. Conclusions

The chemical stability of Ti_3_C_2_ with Al was investigated in the temperature range 500–700 °C in Ar for 1 h. Ti_3_C_2_T_x_ is thermally stable with Al at 650 °C, but it reacts with Al to form Al_3_Ti and TiC at 700 °C. For the Ti_3_C_2_T_x_/Al sample sintered at 650 °C, the Ti_3_C_2_T_x_/Al interface is clean and continuous, without precipitates and amorphous regions. The loss of water molecules and the removal of functional groups of –OH and –O induces a decrease in the interlayer spacing of Ti_3_C_2_T_x_. The present work demonstrates the possibility to prepare Ti_3_C_2_Tx MXene reinforced Al or other metal matrix composites under certain processing conditions.
